# Determining the Margin of Safety for Damaging the Sphenoid Sinus with Nasal Septum Osteotome during Le Fort I Surgery in Young Adults

**DOI:** 10.1155/2018/7465797

**Published:** 2018-11-27

**Authors:** Nesrin Saruhan, Mert Ataol, Mustafa Temiz

**Affiliations:** ^1^Department of Oral and Maxillofacial Surgery, Eskisehir Osmangazi University, Faculty of Dentistry, Eskisehir, Turkey; ^2^Department of Oral and Maxillofacial Surgery, Mersin University, Faculty of Dentistry, Mersin, Turkey; ^3^Department of Oral and Maxillofacial Surgery, Medipol University, Faculty of Dentistry, Istanbul, Turkey

## Abstract

Nasal septum (Obwegeser) osteotome is a basic instrument used for separating the nasal septum and maxilla during Le Fort I osteotomy. If this instrument is placed too high or tilted into the nasal cavity, sphenoid sinus and various adjacent vital structures may be damaged and serious bleeding, neurological complications, or blindness or even death may occur. The aim of this study is to determine the margin of safety for damaging the sphenoid sinus and the adjacent structures with nasal septum osteotome in the young adults: 49 male and 51 female patients between 15 and 25 ages who required a Cone Beam Computed Tomography (CBCT) examination as part of their routine examination. In the study sample consisting of CBCT images, the aimed surgical line, the line between spina nasalis anterior and vomer and the base of sphenoid sinus (undesired line), and tilt angle between surgical and undesirable lines were measured. As the primary outcome of this study, margin of safety for damaging the sphenoid sinus and adjacent vital structures with nasal septum osteotome during Le Fort surgeries in young adults recommended as 5 mm and 12^0^. For this reason the importance of planning with preoperative CBCT before Le Fort I osteotomies has been revealed.

## 1. Introduction

Orthognathic treatment is used worldwide to correct severe dentofacial anomalies with the benefits and risks [1]. Orthognathic surgery has many expected and unexpected complications that cause harm to the patient, occurring either intraoperatively or early or late postoperatively. Some of them are related to nature of surgery and some of them belong to general anesthesia procedures. On the other hand, there are some various rare but severe or life-threating complications [2, 3]. One of major complications is extension of fractures to the pterygoid plate, sphenoid bone, and skull base. It was found to be the cause for devastating complications such as blindness, major bleeding, or even death [4].

One of the basic steps of Le Fort I osteotomy is that nasal septum was detached with forked-type (Obwegeser) nasal septum osteotome, before down fracture of maxilla. This osteotome was used for separating the nasal septum from the maxilla and is positioned on a level with anterior nasal spine and used parallel with the hard palate [5]. At this stage, the area that needs attention is the sphenoid sinus and the adjacent structures [6].

The sphenoid sinus is located in the center of the base of skull. This anatomic sinus was in close relationship by numerous neurovascular structures including cavernous sinus, internal carotid artery, intracranial structures, cranial nerves II to VI, and pituitary gland [7]. Vascular or neurological complications are rare but can lead to permanent disability or even death. So that knowing the details of the anatomy of this region can guide the orthognathic surgeon [8].

Various degrees and directions of pneumatization can occur within the sphenoid sinus. This situation could lead to various extensions, bringing it in close relationship to the cranial nerves, cavernous sinus, internal carotid artery, etc. Some of these structures may underlie and produce bony prominences and related recesses in the sphenoid sinus. Therefore, sphenoid sinus pneumatization should be taken into consideration because the degrees and directions of pneumatization is an important factor that affects the incidence and severity of complications [7]. In a frequently used classification proposed by Hammer and Radberg, sphenoid sinus was divided into three groups based on the extension of pneumatization around the sella turcica in the sagittal plane as conchal, presellar, and sellar types [9]. Conchal type was described as completely missing or minimal sphenoid sinus. Presellar type was described as posterior wall of sphenoid sinus is in front of the anterior wall of the sella. And sellar type was described as posterior wall of sphenoid sinus is behind anterior wall of sella [8]. In the presence of pneumatization, especially in the sellar type which sphenoid sinus pneumatization enlarged, it was considered that the complication incidence rate (due to the thinning of the anterior wall) and the severity of the complication are increased.

Previous studies reported that it is possible to cause sphenoid sinus damage if the nasal septum osteotome is not used carefully [3, 6, 10], but there is no quantitative study of this issue. The aim of this study is to determine the margin of safety for damaging the sphenoid sinus and the adjacent structures with nasal septum osteotome in the young adults.

## 2. Material and Method

This was a retrospective study and the study sample included patients with no history of severe medical illness, craniofacial syndrome, cleft lip or palate, osseous diseases of craniofacial region, and orthognathic surgery. One hundred patients (49 male and 51 female) were enrolled on the study referring to the Eskişehir Osmangazi University, Faculty of Dentistry, who required a CBCT examination as part of their routine examination. The CBCT images were obtained in standing position by using CBCT machine (Planmeca Promax 3D mid, Helsinki, Finland).

**Figure 1:**
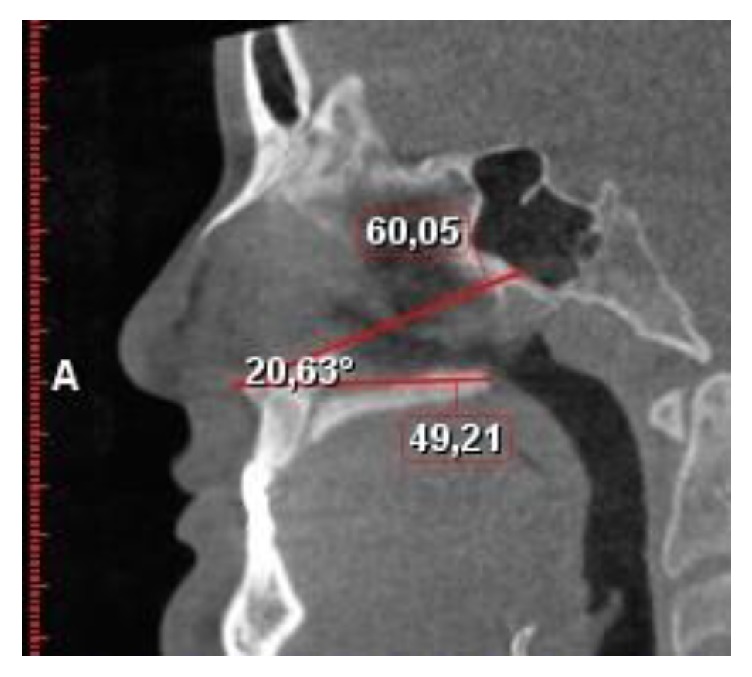
Linear and angular measurements.

In the study sample consisting of CBCT images of patients, two lines and 1 angle were described and prepared as listed:

  Surgical line was between spina nasalis anterior and the junction point of inferior of perpendicular plate of palatine bone
  The undesired line was between spina nasalis anterior and vomer and the base of sphenoid sinus.
  The tilt angle was between the surgical and undesirable lines (Figure 1).

Statistical analysis was performed using IBM SPSS Statistics Version 22.0; Kolmogorov-Smirnov test was used for testing the normality of the distributions. For the intergroup comparisons, Student's T test and independent two-sample test were used. Significance level was set as p < 0.05.

## 3. Results

Study outcomes and descriptive statistics are shown in Table 1. The age range of individuals is 15-25 and the mean age of female individuals including the study is 19 (std. 3.00) and for male individuals is 18.61 (std. 3.25). The mean surgical line for female individuals was 50.64 mm, minimum measurement was 35.65 mm, and maximum measurement was 63.57 mm. The mean undesired line for females was 62.76 mm, minimum measurement was 51.66 mm, and maximum was 70.85 mm. The mean surgical line for male individuals was 52.51 mm, minimum measurement was 41.30 mm, and maximum measurement was 71.83 mm. The mean undesired line for males was 64.25 mm, minimum measurement was 52.22 mm, and maximum was 77.15 mm.

The mean distinction between the surgical and undesirable lines for females was 12.46 mm and for males was 12.66 mm. The minimum distinction for females was 6.98 mm and for males 6.33 mm. The mean tilt angle between the surgical and undesirable lines for females was 16.8 and for males was 18.5^0^. The minimum angle for females was 12.63^0^ and for males 13.91^0^. These results show no statistically significant difference (p > 0.05) in terms of gender, except the tilt angle (p = 0.005).

In present study, sellar type was found as 96% and presellar type was found as 4%. There was no individual which had the conchal type.

## 4. Discussion

In a prospective study with a large series of patients undergoing Le Fort I osteotomy, Kramer et al. [11] reported the overall complication rate to be 6.4%. One of the major complications is cranial complications [12]. Sphenoid sinus traumas or inflammations can cause severe complications that are potentially fatal and visual changes that have been common ranging from 12% to 70% of isolated sphenoid diseases [13]. During the separation of the pterygomaxillary junction, due to the inappropriate forces to the base of the skull via the sphenoid bone and incorrect instrumentation, particularly when nasal septum osteotome is placed too high into the nasal cavity, sphenoid sinus and various adjacent vital structures may be damaged and serious bleeding, neurological complications or blindness, or even death may arise from the operation area [3, 10, 12]. Unwanted fractures extending the cranium could cause cavernous sinus thrombosis or carotid-cavernous sinus fistula; by this way, permanent cranial nerve damage could be observed [6, 12]. These neurological injuries could be isolated or combined [6] and are related to direct or indirect injury. Bony segments resulting from unexpected and unwanted fractures during down fracture of maxilla or with nasal septum or pterygoid osteotome may damage this region. On the other hand, various reasons such ischemia or contusion of a nutrient artery may cause indirect injuries of the adjacent cranial nerves [6, 12]. It is described that direct trauma to the medial aspect of the cavernous sinus bypassing sphenoid sinus may cause neurological complications [14].

Primary outcomes of this study are distinction and tilt angle between surgical and undesired lines. For females, the mean distinction was 12.46 mm and minimum distinction was 6.98 mm. For males, the mean distinction was 12.66 mm and minimum distinction was 6.33 mm. The mean tilt angle between the surgical and undesirable lines for females was 16.8^0^ and for males was 18.5^0^. The minimum angle for females was 12.63^0^ and for males 13.91^0^. According to these results, for a safe surgery, progression of nasal septum osteotome more than 5 mm and 12^0^ should be avoided during Le Fort surgeries in young adults.

The degrees and directions of sphenoid sinus pneumatization are an important factor for the incidence and severity of complications [7]. The sellar type which is the widest pneumatized type was dominant in individuals [15]. In present study, sellar type was found as 96% and presellar type was found as 4%. There was no individual which had the conchal type. It can be said that a large section of the community carries an added complication risk of nasal septum osteotome damage during Le Fort I surgery.

In the evaluation of sphenoid sinuses, conventional radiographs cannot provide accurate information and CT have superseded conventional standard radiography [13]. The use of CBCT in 3-dimensional assessments provides a detailed 3-dimensional morphology of the preoperative structures and could be planned osteotomy lines, measurements, and angles; the image quality is also as good as that of conventional [16].

## 5. Conclusion

As the primary outcome of this study, margin of safety for damaging the sphenoid sinus and adjacent vital structures with nasal septum osteotome during orthognathic surgery in young adults recommended as 5 mm and 12^0^. According to results of this study, if considering anatomic differences and deformities, it is considered that these limitations may be at even more critical levels. For this reason, the importance of planning with preoperative CBCT before Le Fort I osteotomies has been revealed.

## Figures and Tables

**Table 1:** Study outcomes and descriptive statistics.

		Mean	Std.	Minimum	Maximum	p
**Age**	F	19	3.000	15	25	0.537
	M	18.61	3.252	15	25	

**Surgical line (mm)**	F	50.64	4.465	35.65	63.57	0.057
	M	52.51	5.193	41.30	71.83	

**Undesired line (mm)**	F	62.76	4.358	51.66	70.85	0.139
	M	64.25	5.401	52.22	77.15	

**Distinction** ^**1**^ ** (mm)**	F	12.46	4.218	6.98	24.59	0.807
	M	12.66	3.820	6.33	24.30	

**Tilt angle** ^**2**^ ** (degree)**	F	16.80	3.011	12.63	24.09	0.005^*∗*^
	M	18.50	2.702	13.91	27.34	

Std.: standard deviation; *∗*: significant, p < 0.05.

^1^Distinction was obtained by subtracting the length of surgical line from the length of undecided line in mm.

^2^Tilt angle is the angle between surgical and undesired lines.

## Data Availability

The data used to support the findings of this study were provided by corresponding author under license and so cannot be made freely available. Access to these data will be considered by the author upon request, with permission of corresponding author.
